# Developing the "Choosing Health" Digital Weight Loss and Maintenance Intervention: Intervention Mapping Study

**DOI:** 10.2196/34089

**Published:** 2022-10-18

**Authors:** Iga Palacz-Poborczyk, Paulina Idziak, Anna Januszewicz, Aleksandra Luszczynska, Eleanor Quested, Felix Naughton, Martin S Hagger, Sherry Pagoto, Peter Verboon, Suzanne Robinson, Dominika Kwasnicka

**Affiliations:** 1 Faculty of Psychology, SWPS University of Social Sciences and Humanities Wroclaw Poland; 2 Physical Activity and Well-being Research Group, enAble Research Institute, Curtin University Perth Australia; 3 Curtin School of Population Health, Curtin University Perth Australia; 4 Behavioural and Implementation Science Research Group, School of Health Sciences, University of East Anglia Norwich United Kingdom; 5 Psychological Sciences, University of California Merced, CA United States; 6 Faculty of Sport and Health Sciences, University of Jyväskylä Jyväskylä Finland; 7 Department of Allied Health Sciences, The UConn Center for mHealth and Social Media, University of Connecticut Storrs (Mansfield), CT United States; 8 Department of Psychology and Educational Sciences, Open Universiteit Nederland Heerlen Netherlands; 9 Faculty of Health Deakin University Melbourne Australia; 10 NHMRC Centre of Research Excellence in Digital Technology to Transform Chronic Disease Outcomes, Melbourne School of Population and Global Health, University of Melbourne Melbourne Australia

**Keywords:** behavior change, behavior maintenance, behavioral theory, weight loss, overweight, obesity, randomized controlled trial, digital health, within-person design, Intervention Mapping

## Abstract

**Background:**

Digital health promotion programs tailored to the individual are a potential cost-effective and scalable solution to enable self-management and provide support to people with excess body weight. However, solutions that are widely accessible, personalized, and theory- and evidence-based are still limited.

**Objective:**

This study aimed to develop a digital behavior change program, *Choosing Health*, that could identify modifiable predictors of weight loss and maintenance for each individual and use these to provide tailored support.

**Methods:**

We applied an Intervention Mapping protocol to design the program. This systematic approach to develop theory- and evidence-based health promotion programs consisted of 6 steps: development of a logic model of the problem, a model of change, intervention design and intervention production, the implementation plan, and the evaluation plan. The decisions made during the Intervention Mapping process were guided by theory, existing evidence, and our own research—including 4 focus groups (n=40), expert consultations (n=12), and interviews (n=11). The stakeholders included researchers, public representatives (including individuals with overweight and obesity), and experts from a variety of relevant backgrounds (including nutrition, physical activity, and the health care sector).

**Results:**

Following a structured process, we developed a tailored intervention that has the potential to reduce excess body weight and support behavior changes in people with overweight and obesity. The *Choosing Health* intervention consists of tailored, personalized text messages and email support that correspond with theoretical domains potentially predictive of weight outcomes for each participant. The intervention content includes behavior change techniques to support motivation maintenance, self-regulation, habit formation, environmental restructuring, social support, and addressing physical and psychological resources.

**Conclusions:**

The use of an Intervention Mapping protocol enabled the systematic development of the *Choosing Health* intervention and guided the implementation and evaluation of the program. Through the involvement of different stakeholders, including representatives of the general public, we were able to map out program facilitators and barriers while increasing the ecological validity of the program to ensure that we build an intervention that is useful, user-friendly, and informative. We also summarized the lessons learned for the *Choosing Health* intervention development and for other health promotion programs.

**International Registered Report Identifier (IRRID):**

RR2-10.1136/bmjopen-2020-040183

## Introduction

### Background

Worldwide, overweight and obesity are a major public health concern showing continuous increase over the past 4 decades [1]. Excess body weight is a risk factor for multiple chronic health conditions and diseases, including cardiovascular disease, cancer, and type 2 diabetes [2]. Overweight and obesity are also risk factors for severe illness in patients with COVID-19 [3]. The development of programs that support people with overweight and obesity to lose and maintain weight loss is urgently needed [4], especially programs that promote healthy nutrition, physical activity, and health behavior change as a means to lose weight and maintain weight loss. Several evidence-based state-of-the-art weight loss programs exist [5,6]; however, often they are not tailored specifically to the individual. Personalization and tailoring of health promotion programs can increase the cost and complexity of the program [7]; however, it also has multiple advantages.

Intervention tailoring involves adapting the intervention based on specific characteristics of the recipient [8]. A recent systematic review of tailored digital health interventions for weight loss [9] showed that tailored interventions were generally more effective in supporting weight loss compared with generic interventions or waitlist controls. Information can be tailored to the participants in several ways that are reported to vary in effectiveness; most interventions apply *descriptive tailoring,* meaning that participants are provided with information that is tailored based on their responses to a series of questions [9]. For example, participants who report low levels of self-efficacy may receive intervention content that will support them in developing mastery to perform a specific task [10].

An alternative way to tailor the intervention is through *inferential tailoring*, meaning that participants are monitored over a period and information regarding their characteristics, behavioral predictors, and behavioral outcomes is collected (eg, by means of ecological momentary assessment [EMA]) [11,12]. Through the process of longitudinal data collection, the researcher gathers and then analyzes data about the participant (collected using digital technologies) that can provide inferences about what the strongest predictors of relevant outcomes are [13] and, therefore, how the content of the intervention can be most appropriately tailored for them. Inferential tailoring can also account for trends in data and be applied at the time and situation when the intervention is most desirable (eg, by means of just-in-time adaptive interventions [14,15]).

To the best of our knowledge, to date, only 1 study has explored the predictors and outcomes associated with weight loss maintenance in individuals using EMA and N-of-1 designs [16]. The results showed that individuals who lost weight had unique psychological profiles (ie, specific psychological predictors of health behavior change) that could be accounted for in subsequent interventions. However, this study did not use the collected data to personalize the interventions.

To develop personalized interventions, within-person studies exploring weight loss trajectories and changes in cognition and outcomes are needed [17]. Web-based interventions for weight loss and weight loss maintenance in people with overweight and obesity have small to moderate overall effects compared with minimal or control conditions [18]. To move the science forward toward personalized behavioral medicine, new data-driven methods of tailoring and digital health support need to be tested [19]. We now have the opportunity to develop health interventions that are truly individualized and tailored to individuals’ psychological profiles by applying new technologies that support unobtrusive data collection and EMA.

Intervention mapping protocols [20] can be used as a powerful tool to develop effective personalized interventions in a systematic manner. Intervention mapping [21] is a comprehensive framework that can be used to develop new interventions and health promotion programs, adjust existing programs to new contexts and realities [20], and develop implementation strategies [22]. It is an ecological approach that includes active involvement of stakeholders in program development and follows a series of 6 established steps. The steps undertaken are sequential; however, they are also iterative, and intervention developers often move back and forth between the steps to design the most optimal intervention.

Intervention mapping protocols have been successfully applied to design several health promotion programs, including interventions to decrease sedentary behavior [23], improve self-management of type 2 diabetes [24], improve self-care in heart failure [25], prevent risks and hazards in occupational settings [26], and in several other health-related settings. The approach was also applied to design programs that aimed to tackle overweight and obesity in various populations, including children [27,28], pregnant women [29], adolescents [30], adults [31], and workers [32]. Most of the aforementioned health promotion programs used digital technologies; however, none of them included a health behavior change program that was tailored to each individual and based on the participants’ own data via *inferential tailoring*.

### Objectives

In this manuscript, we describe the systematic development of the *Choosing Health* program. An Intervention Mapping protocol guided decisions regarding program objectives, behavior change methods, production, implementation, and evaluation. All decisions made during the Intervention Mapping process were guided by theory [33], evidence [16,34], and our own research undertaken during the intervention design phase (including focus groups, local expert consultations, and interviews). The *Choosing Health* program is a complex health promotion intervention that applies digital technology to collect participants’ EMA data and then use them to provide a tailored intervention. The intervention is aimed at supporting individuals to change their physical activity and nutritional behaviors to ultimately help them lose weight and maintain weight loss. This study aimed to develop a digital behavior change program, *Choosing Health*, following a comprehensive Intervention Mapping protocol.

## Methods

### Study Design

This was an Intervention Mapping study; all study materials, standard operating procedures, and design decisions were documented, and the intervention content was published in the Open Science Framework repository [35]. This Intervention Mapping study resulted in the development of the *Choosing Health* program, which is currently being evaluated through a randomized controlled trial (RCT); this trial was registered at ClinicalTrials.gov (NCT04291482), and the trial protocol was published elsewhere [36].

The Intervention Mapping procedure included 6 steps that comprised several tasks integrating theory and evidence [20]. The completion of all the steps served as a blueprint for designing, implementing, and evaluating the *Choosing Health* intervention based on theoretical, empirical, and practical information. The 6 steps and related tasks of the Intervention Mapping process are described in the following sections and summarized in Multimedia Appendix 1 [9,18,33,36-38].

### Step 1: Needs Assessment

In step 1, we established the planning group (ie, study authors) and conducted a needs assessment to create a logic model of the health problem (Multimedia Appendix 2). The intervention planning group guided the design, implementation, and evaluation of the *Choosing Health* program. This group consisted of 11 key stakeholders representing a variety of backgrounds supported by public representatives, including those representing the program target group (ie, people with overweight and obesity), nutrition and physical activity experts, health care practitioners, and implementers of the program (ie, individuals who could support future rollout of the program). The characteristics of various stakeholders are further described in the Step 4: Intervention Production section. The planning group assessed the issue of overweight and obesity in Poland [39], relevant behaviors, environmental factors, and their associated changeable determinants in the population (ie, individuals with overweight and obesity who require relevant support to lose weight and maintain weight loss). By means of a scoping review, we researched and described overweight and obesity and their impact on quality of life, stating environmental and behavioral changeable determinants. We researched and described the context of the intervention, including contextual factors, community and setting, and defined program goals.

### Step 2: Identifying Objectives

In step 2, we created a logic model of change (Multimedia Appendix 3) defining specific program outcomes and objectives. Following the Intervention Mapping protocol, the planning group worked collaboratively to create a matrix that combined performance objectives (rows) and relevant changeable determinants (columns), listing in the cells specific change objectives. For each change objective, we specified who and what would change as a result of the proposed intervention, setting the foundation for the *Choosing Health* intervention.

### Step 3: Intervention Design

Step 3 consisted of generating theory-based program themes, components, scopes, and sequences. The planning group chose theories that were relevant to the program and decided to include 5 theoretical themes from a recent theory review of behavior change maintenance as underpinning the program [33]. On the basis of the change objectives and determinants identified in the logic model, we also selected change methods and behavior change techniques (BCTs) underpinning the program (which are described in the protocol [36]). Theoretical themes were then mapped to specific BCTs [40], which are also described in the trial protocol [36]. For instance, the theoretical theme of habit formation was supported by specific BCTs such as performing the same behavior in the same context and adding cues to the environment (eg, setting reminders to exercise and putting fresh fruit and vegetables in the lunch pack) so that the context elicits the behavior. We also selected methods of delivery of the program (ie, practical applications to deliver change methods). The planning group chose digital health delivery via text messages, emails, and an intervention book (or e-book) to ensure that the proposed intervention was scalable if proven effective.

### Step 4: Intervention Production

The aim of step 4 was to develop and refine the program structure and organization, preparation of program materials—including drafting theory- and evidence-based messages (emails, text messages, and book)—and program protocols. During this stage, we pretested and refined the materials through focus groups, expert ratings, and interviews.

#### Focus Groups

We conducted 4 focus groups (framed as user engagement workshops, of which 3/4 (75%) were conducted face-to-face and 1/4 (25%) were held on the web because of the COVID-19 pandemic) between November 2019 and May 2020. The focus group participants were recruited through Facebook (event advertisements posted on health-related pages) and through websites listing local events. They were also advertised through newsletters (of the university and of local health-related organizations) and posters placed in community venues and the university. Representatives of the general population (n=40), including some people with overweight (10/40, 25%; 8/10, 80% women and 2/10, 20% men) and obesity (6/40, 15%; 4/6, 67% women and 2/6, 33% men), took part in the focus groups to discuss the project’s rationale, aim, proposed format, and materials. The focus group participants’ mean age was 31.55 (SD 13.15, range 19-65) years, and 22% (9/40) men and 78% (31/40) women took part, with most having a high school education (21/40, 52%) and some having higher education (11/40, 28% Bachelor of Arts or Bachelor of Science and 8/40, 20% Master of Arts or Master of Science. Their average BMI was 24.09 (SD 6.26, range 16.94-34.09; 1/40, 2% of the participants specified their height but not their weight).

The focus group participants assessed and rated the sample intervention materials to assess their clarity (on a scale of 1=unclear to 10=clear), attractiveness (on a scale of 1=unattractive to 10=attractive), and informativeness (on a scale of 1=uninformative to 10=informative). They rated some of the emails (25/96, 25%) and text messages (340/757, 44.9%). Materials were discussed within the group, and the pros and cons were elaborated on. The focus groups were audio recorded, transcribed (by PI), and verified (by IPP), and the transcripts were analyzed verbatim using the framework method [41] in NVivo software (version 12; QSR International) [42]. After familiarization with the transcripts, the first coder (IPP) generated initial themes and indexed the codes in a preliminary framework. These codes and preliminary themes were discussed with the second coder (PI), who independently coded 50% of the transcripts and provided feedback on the themes. The final set of themes was generated using an iterative approach, and all disagreements were discussed with a third researcher (DK) until a consensus was reached.

#### Expert Rating

The full set of intervention materials, including 109 emails and 759 text messages, was pretested with psychology, physical activity, and nutrition experts (n=12). The experts were recruited through the researchers’ network as well as through web-based message boards and Facebook groups for professionals with relevant expertise. The experts were Polish, based in Poland, and they reviewed materials written in Polish. The experts had a mean age of 30.42 (SD 10.9, range 24-64) years and were 8% (1/12) men and 92% (11/12) women; 33% (4/12) had an MSc in Nutrition, 33% (4/12) had an MSc in Psychology, 25% (3/12) had an MSc in Public Health, and 8% (1/12) had a PhD in Health Sciences (including physical activity background). All text messages were assessed by at least two experts who rated the content using the same measures as the ones used during the focus groups to assess content attractiveness and informativeness and, in addition, emotional reactions (*How did it make you feel?* on a scale of 1 indicating negative reactions to 10 indicating positive reactions).

The experts were asked to assign each text message to relevant theoretical domains, with clear definitions of each domain provided (eg, habits, stress, and obstacles). The experts were asked to indicate their first, second, and third choice for the domain that the message aligned with. The experts did not have to have any background in behavioral science to assign messages to theoretical domains as clear definitions were provided and examples were given. They also provided additional open-ended comments if they had any feedback or reflections regarding specific text messages or emails. The experts completed this task in a Microsoft Excel form in their own time. The theoretical domains and definitions of the theoretical constructs were based on a comprehensive theory review [33].

#### Interviews

We also asked 11 representatives of the general population (n=6, 55% men and n=5, 45% women; mean age 39.27, SD 16.32, range 18-72 years) to evaluate the intervention book or e-book (our program participants had a choice between a physical book and an e-book). Interviewed participants were recruited through the researchers’ networks. Each person read through the whole book and, by means of unstructured interviews (conducted by IPP), provided feedback on content, comprehensibility, user-friendliness of the design, and inclusiveness. The key points from each interview were summarized and noted by the interviewer, and the book was revised in line with the suggestions given.

The materials (emails, text messages, and book) were iteratively revised by 4 members of the project team (IPP, PI, DK, and AJ) and continuously adapted based on insights from the focus groups, interviews, and study experts. During the intervention content development stage (June 2020-August 2020), the core team (IPP, PI, DK, and AJ) met 11 times; each meeting took 2 to 3 hours, approximately 30 hours in total. The *Choosing Health* program protocol, including frequency, intensity, and sequence, was also discussed and agreed upon. All materials were developed in Polish and later translated into English and published on the Open Science Framework website. All study measures were forward and backward translated [43] if language-specific versions of the questionnaires were not available. All questions were adjusted for culture- and language-specific appropriateness and piloted with Polish speakers (n=15), with changes made in line with the feedback received. The project team members (IPP, PI, AJ, and DK) met 3 times (approximately 12 hours in total) to finalize the translation and adaptation of the questions and measurement tools for the trial (October 2019-November 2019).

### Step 5: Implementation Plan

In step 5, we defined the intervention adaptation, implementation, and sustainability plan developing matrices defining change objectives to promote *Choosing Health* program adaptation and use. These objectives were operationalized forming theory-informed plans for intervention adaptation and implementation [44,45]. Through discussion, the planning group identified potential program users (adopters, implementers, and maintainers) considering both the initial program test (RCT) and if the program was to be widely implemented. Behavioral outcomes were defined and linked to the behavioral and environmental determinants. The resulting change objectives for program use were used to map the intervention for potential adopters, implementers, and maintainers designing the intervention implementation plan, which is further described in the Results section.

### Step 6: Evaluation Plan

In the final step, we planned how to best evaluate the program effects, costs, and processes. A specific evaluation plan was developed by the core planning group, and the trial protocol was published [36]. We defined the mechanisms of intervention effectiveness informed by the previous Intervention Mapping steps. Following the Intervention Mapping protocol [20], we listed the effect, cost, and process evaluation research questions that are listed in the protocol [36]. We developed indicators and measures of success by defining study measures, measurement points, and thresholds for effectiveness based on the previous literature [17]. The intervention is currently ongoing through an RCT with an embedded N-of-1 study and ongoing cost and process evaluations.

### Ethics Approval

Ethics approval was granted by the Faculty of Psychology, SWPS University of Social Sciences and Humanities, Poland (approval 03/P/12/2019).

## Results

In this study, we used the Intervention Mapping approach following the aforementioned steps (Multimedia Appendix 1). In step 1, a needs assessment was used to define the problem—namely, high levels of obesity and overweight in Poland (reaching >53.3%) [39,46] and the need to design effective, cost-effective, and scalable programs that can support people in weight loss and subsequent weight loss maintenance. The impact on quality of life was prominent, with people with overweight and obesity reporting lower physical and mental health [39,46]. Several environmental and behavioral determinants were described and listed, including limited access to weight loss programs, the high cost of weight loss programs, obstacles to accessing healthy foods (eg, perceived as more expensive than unhealthy foods), and obstacles to engaging in physical activity (eg, perceived lack of time and limited access). Contextual factors included personal, family, work, and broader community influences, which could both enable and hinder behaviors conducive to weight loss and weight loss maintenance. The variety of determinants and contexts that needed to be considered pointed toward the need for a highly personalized and cost-effective program.

In step 2, the program’s objectives were specified—namely, to develop a program that could support self-guided personalized weight loss, including behavior changes in physical activity, nutrition, and prompting psychological changes (in motivation, habits, self-regulation, resources, and context). The list of combined performance objectives and relevant changeable determinants included the following: individuals who complete the program will need to complete 2 key phases to lose weight and maintain weight loss. First, we need to learn about their individual predictors of weight loss and weight loss maintenance (therefore, we will encourage program users to self-monitor their determinants—theory-based constructs including motivation, habits, self-regulation, resources, and context). Subsequently, we will intervene on the strongest predictors of behavioral outcomes, providing relevant intervention content. For each determinant, we mapped the corresponding theoretical explanations and techniques [36]. We predicted that there are several changeable determinants that are relevant to each program user; however, each user is likely to have a different profile of determinants that are the most predictive of weight change and maintenance.

In step 3, techniques fitting the problem and objectives were chosen. On the basis of theory and evidence, we divided our intervention into 5 conceptual domains (maintained motivation, habit, self-regulation, resources, and environmental influences) and, within these domains, suitable BCTs were identified [36]. For instance, to support habit formation, we prompted rehearsal and repetition of the behavior in the same context so that the context elicited the behavior. We mapped out BCTs to each domain and operationalized them in intervention materials, including text messages, emails, and e-book.

In step 4, we conducted focus groups to refine the intervention content. A total of 40 participants took part in the focus groups, and the key themes discussed were analyzed and divided into 2 groups of themes: intervention content and form of program delivery. The first theme had three main subthemes: (1) the participant being an active agent in the change process, (2) inclusivity of the information provided, and (3) problem-solving. The second theme also had three main subthemes: (1) ensuring that the content was informative, (2) unambiguity of the provided information, and (3) including direct actionable messages. In Table 1, we include *lessons learnt* from the focus groups in relation to intervention content and form and direct quotes from focus group participants that align with different themes and subthemes.

The focus group participants’ mean scores for the proposed text message content were relatively high on a scale of 1 to 10 (mean 8.38; clarity mean 9.27, SD 1.32; attractiveness mean 8.48, SD 1.24; informativeness mean 8.6, SD 1.33); higher scores reflected positive results, and lower scores reflected negative results for each category. These findings were corroborated in the focus group discussions (Table 1).

Experts rated the quality of the text messages as moderately high (mean 7.21; positive emotions mean 6.95, SD 1.41; attractiveness mean 7.23, SD 1.38; informativeness mean 7.47, SD 1.61). All text messages rated below an average of 4.5 across all categories were excluded or adjusted, and text messages that did not fit specific themes were reallocated or adjusted. In total, we excluded 3.7% (28/759) of text messages and 0.9% (1/109) of emails that were considered inappropriate or scored low overall.

In step 5, we identified potential program users as adults with overweight and obesity living in Poland. Initial program users were individuals living in Wroclaw and nearby areas as the initial test of the program (via RCT) required face-to-face assessments to objectively measure weight. Implementers of the program initially included the researchers involved in the program development and research assistants. Future program implementers and maintainers (if the program is proven effective) could include representatives from the government, health care representatives recommending the program, and community representatives. One of the routes to intervention implementation that we are assessing now is wide implementation through the program partner Lifestyle Medicine [47,48]—an organization that provides medical training for health care practitioners educating them on the principles of behavior change and advocating to promote health behavior change in patients and minimize the overmedicalization of people with overweight and obesity. If the intervention is effective, it could contribute to lowering overweight and obesity rates in Poland, resulting in health improvements and cost savings.

In step 6, we generated a plan for cost, effect, and process evaluations. Currently, the program is being evaluated through an RCT assessing between-group effects (intervention vs control), and it is also being evaluated within people looking at the trajectories of change investigated through EMA using an N-of-1 design and inferential tailoring [36]. The resulting program is evidence-based, delivered through technology (text messages, email, and book), and tailored to each participant based on the data gathered through EMA. The evaluation plan follows the principles of process evaluation defined by the Medical Research Council (United Kingdom) following the guidelines for developing and evaluating complex interventions [37].

**Table 1:** Lessons learnt for the *Choosing Health* program and for other intervention developers from the focus groups undertaken (N=40)^a^.

Theme and theme description			Lessons learned		Example quotes
**Intervention content**					
	*The participant being an active agent in the change process*: study participants much preferred the messages that treated them as experts in their own behavior change. Any messages that could come across as condescending or coming from the perspective of a “teacher” or a person who “knows it all” or “knows better” were considered inappropriate.	Study participants need to be treated as equal partners in the behavior change and behavior maintenance process. Understanding of personal needs and preferences is key to providing useful intervention content. Each message needs to contain elements of flexibility (the participant may want to take the suggestion on or not; they do not need to follow the suggestions fully).		Condescending, stereotypical, and negative messages were unacceptable: “Not everyone who carries extra kilograms sits nonstop in front of the TV and eats crisps. We can’t speak to them [intervention users] as if they did not have a clue that a week without the TV or a week without crisps is possible. The worse thing we could do is to look down on them.” [Participant 14, woman, aged 24 years, BMI 29] Intervention aims should be personalized and defined with the study participants: “I would simply ask what are the intentions of this person, what exactly motivates them? Why are they taking part in your program? Probably they want to lose weight but you need to understand other factors too...” [Participant 7, woman, aged 30 years, BMI 20] To many participants, the provided information was not new and often complemented what they already knew and what they had already experienced: “From my own experience I can say that the feeling of hunger is just so personal. I had to relearn to understand when I’m hungry, when I’m full and when I’ve totally overeaten. Since childhood I was ‘trained’ to eat like a horse, to just feel more than full. I had to relearn to eat till I’m almost full, so I feel slightly unsatisfied. Some people still need to learn it and work on it.” [Participant 9, man, aged 47 years, BMI 20] People’s levels of motivation and motivation sources vary, and interventions need to account for that: “Social support is very important but if other people don’t want to support me, they should at least not criticise my choices. I look for support or at least lack of criticism of what I do. Maybe other people find it helpful to be criticised, for me, I find it really demotivating.” [Participant 9, man, aged 47 years, BMI 20] All messages suggesting that physical activity needs to be chosen in line with personal preferences were rated positively: “I really get on board with this, I really like that you suggest that physical activity doesn’t need to be forced, and that I can just pick whatever I like, as long as I am active.” [Participant 39, man, aged 39 years, BMI 25] Most messages need to give the participant some options and choices. The participants prefer to choose what fits their lifestyle and preferences: “I love this message—I like that you say that there is not one type of food that makes someone feel better—one person may like nuts, other one may prefer fish, I just really like how you pointed out that this is all personal.” [Participant 38, man, aged 65 years, BMI 30]	
	*Inclusivity of the information provided*: it was important to tailor messages so that they fit in with people who are of different socioeconomic statuses or different personal circumstances, prefer different leisure activities, and have different health statuses and professions.	The person’s identity needs to be considered when defining intervention content. The information provided needs to be inclusive, especially when discussing social support.		Participants who did not have close family or lived far away from their family felt excluded when reading the messages pointing toward fun social family activities: “Someone who I don’t actually even know, writes to me and says hug a family member, and I’m alone, I do not have any close family, I would get so p***** off, and sorry to phrase it like that, but I would just not continue with this.” [Participant 32, woman, aged 44 years, BMI 28]	
	*Problem-solving*: study participants wanted to receive positive messages that motivated them to problem solve. The superficial approach of “it’s all good” and “you can do it!” was not perceived as helpful. The participants needed some acknowledgment that weight loss is not easy and often comes with barriers and difficulties.	People usually knew what the negative consequences were, and they did not need to be reminded. They needed constructive suggestions for how to best problem solve. Messages based on fear and negative emotions were considered unhelpful.		Participants appreciated messages that emphasized their psychological resources and constructive ways of using self-motivation: “The message I really, really like is this one: ‘Think about the day when you decided to join Choosing Health program! What motivated you to join? Note down thoughts that you had then.’ I really liked this message coz people often undertake challenges and then half way through they forget why they actually doing it. The motivation is gone, and sometimes it’s enough to just remind someone why are they doing it. Remembering your past success, can really reinforce your motivation and help you look more positively towards the future.” [Participant 24, woman, aged 24 years, BMI 26] The messages that described unpleasant situations, evoked negative emotions, and reminded the participants of some negative past events but did not include any actionable solutions that needed to be avoided: “Imagine, I’m in a good mood, having a really good day, everything going well and then I’m getting one of your messages, this one, it says ‘consider what’s causing stress in your life and think about how you could tackle it and change it’—So now what? I’m doing my exercise, drinking water, I eat healthily and now what? I’m stressing thinking oh dear God...my husband, all the debt I have...” [Participant 29, woman, aged 34 years, BMI 28] The participants rated positively the messages that encouraged them to self-monitor and pointed them toward the strategies that they could implement immediately to improve: “I really like these messages that said that I should write down certain things, note what motivates me, and note what my goals are. That was great, a systematic way of doing things, if I write it down, I will remember it. If I read your text and I’m on the go, I may remember it but I may not...” [Participant 30, woman, aged 37 years, BMI 21]	
**Intervention form**					
	*Ensuring that the content is informative*: the participants really appreciated the fact that the intervention was evidence-based. The expectations were high in terms of providing the most recent psychological knowledge. The participants wanted to receive fresh and novel content, and they wanted to develop their own knowledge.	Just the fact that participants receive messages may be motivating, but this motivation is not long-lived if the content is not informative. To maintain participants’ engagement, they need to receive evidence-based, state-of-the-art, engaging, and original content. People do not want to be overloaded with the information.		It is important that the participants learn something new that they did not know before: “I really liked it because the information provided was interesting and some of it was surprising for me, especially when you elaborated on different causes of stress. It was clear, it made me feel good, I simply learnt new information.” [Participant 36, woman, aged 25 years, BMI 17] People want to read evidence-based information: “Some of the messages were just too simple. I don’t want to sound big-headed but for me that was just way too simple. I would add (to the emails) at least few lines describing some background evidence, at least something showing that it’s actually based on scientific evidence.” [Participant 26, man, aged 24 years, BMI 20] The intervention should encourage them to learn more: “Maybe you could encourage people to be more conscious and to learn more: ‘Check if what you think is healthy, is actually healthy and good for you?’ [...] A while ago I went to the shops, I had some time and I saw minced meat. I usually don’t buy that type of meat, and then I read what’s on the label, and I couldn’t believe it! [...] Maybe you could consider that people need to seek to educate themselves a bit more.” [Participant 21, woman, aged 34 years, BMI 34] The intervention should include valuable messages without sounding superficial: “I just have a general suggestion—for me messages that include phrases ‘healthy food,’ ‘balanced diet,’ ‘balance’ sound a bit superficial, as people don’t really know what that means.” [Participant 26, man, aged 24 years, BMI 20] Explaining psychological and physiological mechanisms related to weight loss was always welcome, especially if communicated in a clear way (but only in emails as SMS text messages were perceived as too short to clearly communicate the meaning and dependencies): “I was positively surprised to read the email ‘What stress actually is?’ [...] This message had a lot of important, easy to digest info, stress is so common these days so I was glad to read more on this topic.” [Participant 36, woman, aged 25 years, BMI 17] Psychological evidence was expected, and the participants wanted to learn more about the mechanisms of action and behavior change techniques: “So where are all the psychological aspect here? You say monitor eating to not overdo it, but how am I meant to do that?!” [Participant 32, woman, aged 44 years, BMI 28]	
	*Unambiguity of the provided information*: messages should have a clear meaning and preferably cover only 1 topic at a time.	When the message includes multiple topics, it is more difficult to understand, reflect on, and implement.		The participants did not perceive it helpful when healthy eating, physical activity, education, and social support were all mentioned in one message: “For me it is problematic that you talk about healthy eating and physical activity. Someone may be concentrating hard on eating healthily but not really on improving physical activity. [...] I would probably just emphasize one or the other as tackling both at the same time is hard.” [Participant 7, woman, aged 30 years, BMI 20]	
	*Including direct actionable messages*: participants did not appreciate idioms or references to literature, culture, or pop culture. The use of humor was controversial and had very diverse reception.	Study participants preferred direct, clear, and actionable messages.		Participants did not appreciate “mental shortcuts,” and every change of topic in the message had to be clearly announced to them: “This message about feeling grateful—I completely don’t get what’s the relationship between feeling grateful and losing weight or improving health.” [Participant 38, man, aged 65 years, BMI 30] Idioms and messages including humor were negatively received. Losing weight and maintaining weight loss are often perceived as sensitive topics, and the use of humor is often considered inappropriate: “...again you are using an idiom here—and I’m fairly sure that not everyone is able to understand it in this context.” [Participant 40, man, aged 65 years, BMI 32]	

^a^Interview data were analyzed with the aim of improving intervention content and form (ie, look and feel, intensity, and sequence) so that the main themes were predefined before the analysis process. The subthemes emerged from the discussions and data analysis.

## Discussion

### Principal Findings

The overall aim of this Intervention Mapping study was to inform the *Choosing Health* weight loss program by applying existing theory, evidence, and principles of public engagement throughout the planning process. The Intervention Mapping protocol steps were closely followed to ensure that the intervention was useful, user-friendly, and designed with the users to ensure the clarity, attractiveness, and positive reception of the proposed program. The focus groups, expert consultations, and interviews showed that the program content and proposed format were rated highly, and the elements that did not meet a specific threshold (4.5 out of 10) were adjusted in line with feedback or omitted. Active involvement of individuals from the target group of the *Choosing Health* intervention—namely, people with overweight or obesity—enabled the specification of the key needs and wants of the recipients of the intervention. The materials were produced iteratively and sequentially, and the mode of delivery was thoroughly discussed with the potential users to ensure the feasibility of the proposed program.

In relation to the intervention content, the main results were that program participants need to be actively involved in the change process, which aligns with theory [49] and previous interventions [50]. The information provided needs to be inclusive and encourage the participants to actively problem solve while they are changing their behavior and maintaining it in the long term [51]. In terms of the format of the program, the key results were that it needs to be informative and unambiguous and include direct and actionable messages, aligning with other recommendations for the development of health behavior change programs [52,53]. Previous studies that also gathered EMA data on daily predictors of weight loss [16] did not use inferential tailoring to provide health behavior change advice and information. This will be the first study that uses longitudinal data to then provide tailored support.

This study has several strengths. The key strength is the use of the thorough and rigorous Intervention Mapping protocol that served as a tool and provided us with vocabulary to comprehensively map out and plan the proposed intervention [20]. Designing interventions iteratively with the users and using a variety of study methods ensures that the interventions have high ecological validity [54]. The intervention was designed by the core group (the study authors) in close collaboration with public representatives and field experts (eg, nutritionists and physical activity experts) to increase the ecological validity of the proposed program. Engaging the target audience in the intervention design ensures that the programs are suitable and useful and that they target relevant determinants [55]; it also ensures that we account for diversity in the participant population [56]. The intervention content was designed to be tailored to specific theoretical domains that were predictive of effects (that will be assessed by participants through EMA to define the strongest predictors of outcomes). We consulted the experts and asked them to allocate each element of the intervention content to a specific theoretical domain to ensure that the content fits the theoretical domains. This validation ensured that we targeted the correct determinants that are the strongest predictors of outcomes for each individual.

The study limitations include lack of involvement of some key stakeholders in the planning group. Namely, representative policy-makers from the local or national government and IT did not participate in the planning group. To ensure scalability and long-term maintenance of the proposed program, it would need to be integrated with existing intervention programs or policies operating within the health care system or local communities [57,58]. The planning group met with representatives from the Ministry of Health of Poland, who initially expressed support to promote the project and implement it at a national scale if proven effective through the nationwide health care and health promotion website [59]. However, following structural and personnel changes in the national government, the plan was no longer feasible to implement. Liaising with the Ministry of Health during the COVID-19 pandemic also proved difficult. Other studies and health promotion programs emphasize how valuable it is to engage policy-makers in the intervention development and implementation processes [57,58], and we are hoping to meaningfully engage with them in the future. To scale the intervention (if effective) and make it accessible to people in Poland, a national body (government and health care sector) needs to endorse the intervention and embed it within the existing support structures. The COVID-19 pandemic has emphasized the need for self-guided remote support to improve health.

The proposed intervention has specific components that combine different technology aspects—data harvesting via EMA, automated text messaging, automated emails, and book; therefore, this intervention could further benefit from the active involvement of technology developers, data scientists, and computer programmers. The researchers working on the project designed the technology interface using existing components (eg, automated messaging systems). However, to further enhance the scalability of the intervention, the engagement of computer scientists would allow us to implement more sophisticated data analytics and intervention setup methods. Future interventions need to include automated machine learning algorithms that would allow for the analysis of data in real time and automated setup of the intervention to improve efficiencies and reduce the resources needed [60,61]. Machine learning is a valuable and increasingly necessary tool for health promotion and for the modern health care system [62], and it should be applied in future personalized interventions.

In Poland, the prevalence of overweight and obesity is higher in men than in women (46.8% vs 32.2% for overweight, respectively, and 20.1% vs 18.1% for obesity, respectively); however, the sample recruited for the focus groups comprised predominantly women (31/40, 78%) and a predominantly normal weight BMI category (24/40, 60%). In the ideal scenario, most of the focus group participants would have been men, and most or all participants would have been overweight and obese. Specific challenges, including social stigma and stereotypes associated with dieting and weight loss programs, played a role in recruiting a more representative sample of the user population. However, we have explored whether there are any differences among the opinions and feedback given by men and women and also by people who fall into BMI categories below and above 25, and we have not found any pronounced differences.

The key take-home messages from our *Choosing Health* Intervention Mapping study were (1) involving several types of stakeholders as early as possible in the Intervention Mapping process, (2) iterating the intervention with various groups of stakeholders and learning from the incoming evaluation data, and (3) allowing for flexibility in health promotion programs. As the intervention was designed to be delivered on the web, the COVID-19 pandemic did not have an impact on the delivery of the intervention; however, it has affected study data collection that was initially intended to be conducted face-to-face. In addition, one of the focus groups had to be conducted on the web. Several research teams working worldwide are facing similar challenges, and specific technology solutions are being developed to support these teams in data collection during the pandemic [54,55]. Currently, developing health promotion programs that can be fully delivered on the web is important and needed.

### Conclusions

We developed a comprehensive weight loss and maintenance intervention targeting important behavioral and contextual determinants. The development of the intervention followed comprehensive steps of the Intervention Mapping process and was grounded in theory and relevant literature. Future evaluation studies will investigate the program effectiveness, cost-effectiveness, and process and further analyze the relevance and utility of the specific program components. The findings from this study may be particularly useful for other intervention developers who are also planning to design and implement personalized digital health weight loss interventions targeting behavioral nutrition, physical activity, and health behavior change.

## Appendix

Multimedia Appendix 1 The 6 steps of Intervention Mapping undertaken during the Choosing Health program development combining methodologies and results.

Multimedia Appendix 2 A logic model of the health problem.

Multimedia Appendix 3 The logic model of change.

## Abbreviations

BCT: behavior change technique
EMA: ecological momentary assessment
RCT: randomized controlled trial

## Data Availability

The data sets generated and analyzed during this study are available in the Open Science Framework repository [35].
