# Supplementary material for: Developing the "Choosing Health" Digital Weight Loss and Maintenance Intervention: Intervention Mapping Study
Source: J Med Internet Res. 2022 Oct 18;24(10):e34089. doi: 10.2196/34089 (PMC9627465; doi:10.2196/34089)
Supplement: Multimedia Appendix 1 [file jmir_v24i10e34089_app1.docx]

**Supplementary Material 1**

The six steps of Intervention Mapping undertaken during the *Choosing Health* program development combining methods undertaken and results.

|  | **Steps** | **Summary of the activities undertaken during each step and their results** |
| --- | --- | --- |
| Ongoing evaluation | **Step 1.**  The Logic Model of the Problem | 1. - Planning group was established and we mapped out project plans and jointly applied for program funding. 2. - We conducted a needs assessment to create a logic model of the problem, namely overweight and obesity rates increasing, and lack of evidence- and theory- based personalised programs that are cost effective and scalable. 3. - We described the context for the program, including the population (people with overweight and obesity), setting (living in Wroclaw, Poland and nearby as a test-bed for the intervention), and community (recruitment through several communities, mainly online via social media). 4. - We stated program goals (main goal being research of effective and scalable personalised intervention to reduce overweight and obesity in the specific population, and if effective, roll out of the program). |
|  | **Step 2.**  The Logic Model of Change | 1. - We stated expected outcomes for behaviour and environment, including increasing physical activity, modifying nutrition behaviours, changing psychological variables (such as motivation). Environmental factors included: adding objects to the environment, and providing social support. 2. - We specified performance objectives for behavioural and environmental outcomes, including engagement with the intervention and following the advice provided by the tailored support. 3. - We selected determinants for behavioural and environmental outcomes, including motivation, habitual behaviours, self-regulation, resources and contextual factors (including physical and social environment). 4. - We constructed matrices of change objectives listing objectives and determinants. 5. - We created the logic model of change with a key assumption that if the participants provide us sufficient data for intervention tailoring, we will be able to provide personalised digital health intervention that will target specific determinants of outcomes and subsequently improve outcomes leading to weight loss. |
|  | **Step 3.** Program design | 1. - We generated program themes, components, scope, and sequence, including the sequence of emails and text messages send to the participants. 2. - We chose theory- and evidence-based change methods closely based on theoretical themes from the Theory Review [33], and from evidence from systematic reviews [9,18,62]. 3. - We selected practical applications to deliver change methods including digital health intervention delivery. |
|  | **Step 4.** Program Production | 1. - We refined program structure and organisation based on insights from the focus groups (user-design workshops) and through expert consultations and scoring. 2. - We prepared plans for program materials and worked closely with a professional designer to develop program materials that were subsequently piloted with the potential users. 3. - We drafted messages, materials, and protocols that were also revised through the focus groups (user-design workshops, *N*=40) and through expert consultations and scoring (*N*=12). 4. - We pretested, refined, and produced materials, presenting them to the potential users and thoroughly revising the content. |
|  | **Step 5.** Program Implementation Plan | 1. - We identified potential program users and recruited them to participate in the intervention (via RCT) with embedded N-of-1 study. 2. - We stated outcomes and performance objectives for program use and published a study protocol [35]. 3. - We constructed matrices of change objectives for program use and designed plans for the implementation of the intervention. |
|  | **Step 6.** Program Evaluation Plan | 1. - We noted effect and process evaluation questions and measurement methods to assess them. 2. - We developed indicators and measures for assessment and defined measurement points at the individual and group level. 3. - We defined the evaluation plan following process evaluation principles [46]. 4. - We will complete the evaluation plan once the trail is executed. |

*Note.* The steps are described sequentially; however, the process was iterative and we often progressed back and forth between the Intervention Mapping steps.
