# Supplementary figures and images for: Developing the "Choosing Health" Digital Weight Loss and Maintenance Intervention: Intervention Mapping Study
Source: J Med Internet Res. 2022 Oct 18;24(10):e34089. doi: 10.2196/34089 (PMC9627465; doi:10.2196/34089)

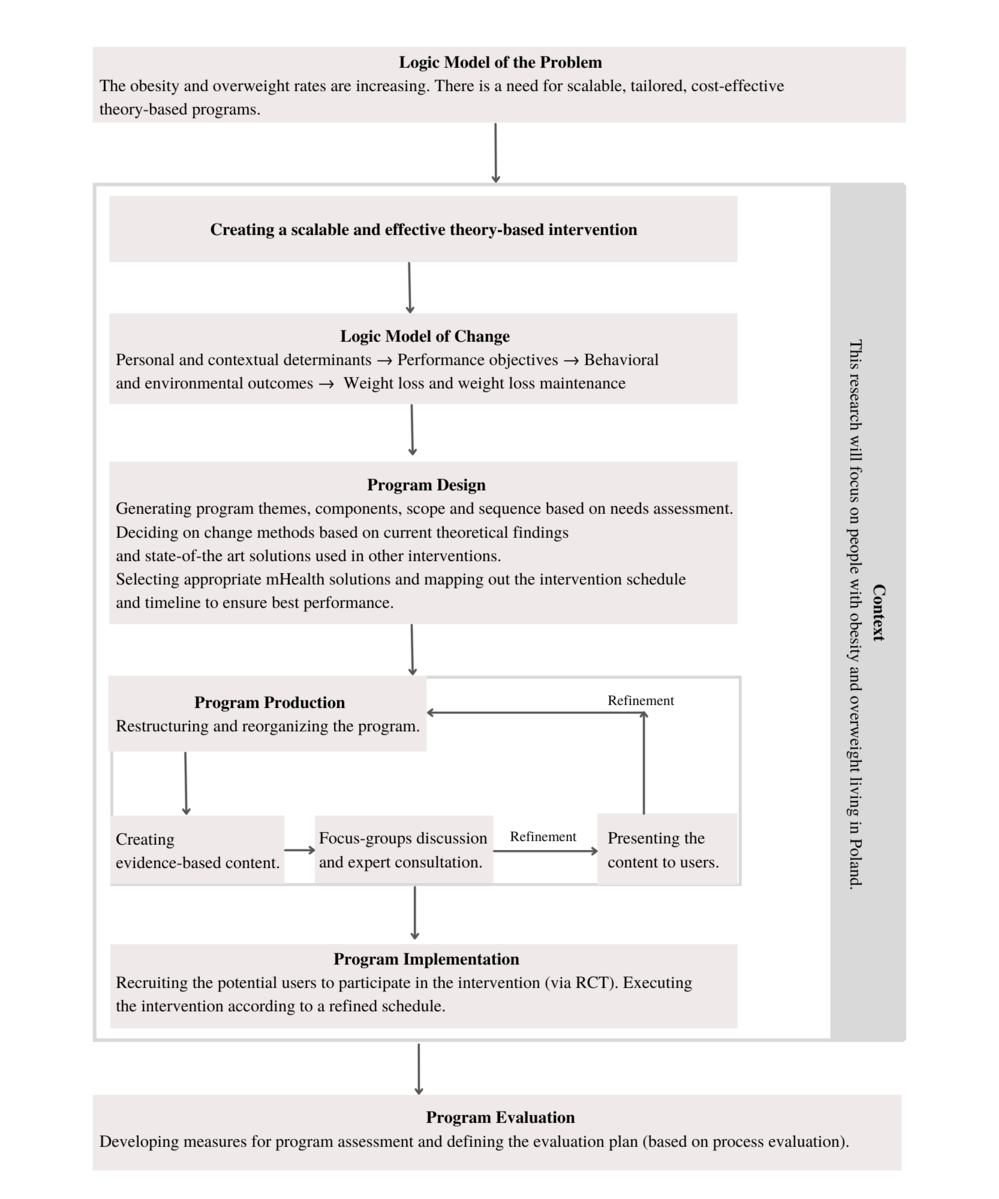

Supplement: Multimedia Appendix 2 [file jmir_v24i10e34089_app2.png]

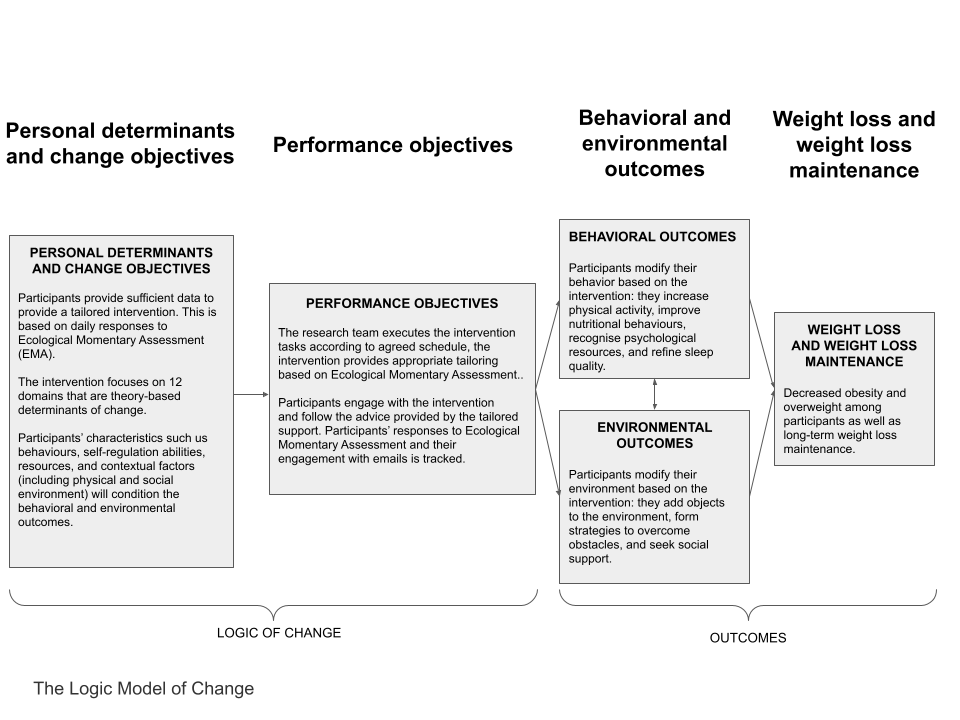

Supplement: Multimedia Appendix 3 [file jmir_v24i10e34089_app3.png]
